# Osteoid Osteoma of the Basilar Border of the Mandible: A Diagnostic Dilemma

**DOI:** 10.1155/2022/2179877

**Published:** 2022-12-14

**Authors:** Rym Kammoun, Imen Chaabani, Sonia Ghoul, Touhami Ben Alaya

**Affiliations:** ^1^Laboratory of Histology and Embryology, Faculty of Dental Medicine of Monastir, University of Monastir, Monastir, Tunisia; ^2^ABCDF Laboratory for Biological Clinical and Dento-Facial Approach, University of Monastir, Monastir, Tunisia; ^3^Department of Radiology, University Dental Clinic, Monastir, Tunisia; ^4^Unity of Bioactive Natural Substances and Biotechnology, Faculty of Dental Medicine, University of Monastir, Monastir, Tunisia

## Abstract

*Introduction*. Osteoid osteoma is a benign osseous tumor characterized by an excessive formation of unmineralized bone matrix. The aim of this study was to present, through a case report, the clinical and radiological manifestations of osteoid osteoma affecting the left basilar border of the mandible. *Observation*. A 30-year-old male patient presented with left mandibular pain of unknown etiology. The chief complaint was mandibular pain accentuating mainly at night, originating in the left basilar border, and radiating to the whole mandibular hemi-arch. Extraoral examination revealed a small, bone-consistent, and slightly painful swelling. Computed tomography scan revealed a well-limited, infracentimetric, and mixed osteolytic image with central nidus and peripheral osteocondensation. Histological examination revealed osteoid osteoma. Surgical excision of the lesion was performed, and the pain stopped immediately. *Discussion*. This benign tumor presents many clinical and radiological features similar to other lesions. To differentiate osteoid osteoma from these other bone pathoses, practitioners should have a clear concept and keen observation skills. Confrontation of the clinical, radiological, and anatomopathological data is therefore essential to establish the correct diagnosis and to determine the appropriate treatment plan.

## 1. Introduction

Condensing images of the jaw bones represent a heterogeneous group of lesions with several possible diagnoses. Some are specific to the jaws, whereas others can be found throughout the skeleton.

Osteoid osteoma is a benign bone tumor, with prevalence in the jaws accounting for less than 1%. This entity accounts for 3% of all primary bone tumors and 10% of benign bone tumors [1]. It occurs more frequently in the long bones of the lower limbs than in the long bones of the upper limbs. It can also develop in the axial skeleton. It rarely occurs in the jaws, with the mandible more commonly affected than the maxilla [2]. Osteoid osteoma is more frequent in adolescents and young adults, with the peak of incidence being between 20 and 30 years of age. Typically, it is more common in male than female subjects [3].

This benign bone tumor is characterized by an excessive formation of unmineralized bone matrix, called osteoid. It has a small, oval, or round centrally located area, called nidus, composed of osteoid and trabeculae of newly formed bone, deposited within highly vascularized connective tissues and surrounded by reactive sclerotic cortices. This tumor has a limited growth potential reaching a maximum size of just 1–2 cm [4].

The true nature of this lesion is still unknown. It is considered as a low growth neoplasm or an inactive neoplasm by some authors and as the consequence of a trauma or an inflammatory process by others [5].

Lack of knowledge concerning the etiopathogenesis of this tumor and its confusion with similar bone lesions, such as odontogenic lesions, make its diagnosis difficult [6]. Furthermore, because it is a slow-growing tumor, it usually manifests at a late stage. Thus, its exploration during the initial stages is often incidental due to the lack of clinical manifestations [3].

Proper diagnosis and a well-established treatment plan are fundamental because of the specificity and scarcity of this tumor. Diagnosis is greatly made based on the clinical features and radiological imaging, and it is confirmed by the histopathological examination. Confrontation of the clinical, radiological, and anatomopathological data is, therefore, essential to make the correct diagnosis and to subsequently establish the appropriate treatment plan.

The aim of this study was to present, through a case report, the clinical and radiological manifestations of osteoid osteoma affecting the left basilar border of the mandible.

## 2. Case Presentation

A 30-year-old male patient, in good general condition, was referred by his treating dentist to the Department of Oral Medicine and Surgery for left mandibular pain of unknown etiology.

The patient's chief complaint was mandibular pain originating in the left basilar border and radiating to the whole mandibular hemi-arch. He had undergone endodontic treatment on the left mandibular second molar (tooth #37). On extraoral examination, a small, bone-consistent, and slightly painful swelling was noted on palpation of the left basilar border. The patient reported that the pain accentuated mainly at night. No labiomental hypoesthesia was noted, and the rest of the extraoral examination was normal. Intraoral examination revealed the presence of a temporary coronal filling on the left mandibular second molar (tooth #37). Axial and transverse percussions were painless. No swelling was noted on palpation of the vestibular bottom.

**Figure 1:**
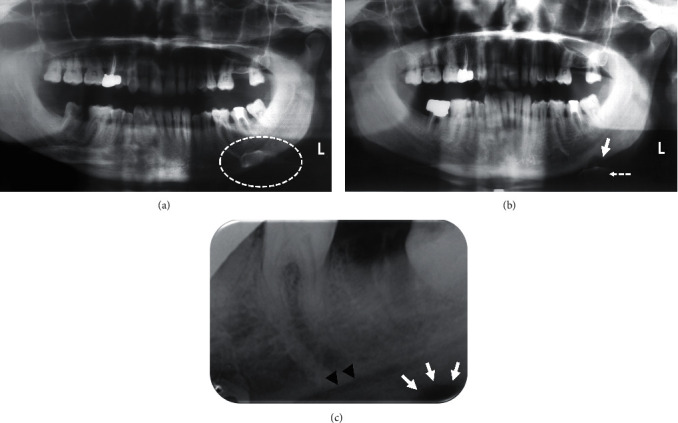
Standard radiological assessment. (a) Initial panoramic radiograph showing superposition of the hyoid bone on the left mandibular basilar border (white dashed circle), masking the osteolytic image. (b) Panoramic radiograph repeated with the patient in a slightly hyperextended position, allowing a lower projection of the hyoid bone (dotted white arrow), and a good visualization of the osteolytic image of the left basilar border (white arrow). (c) Retroalveolar radiograph performed after extraction of the tooth #37 showing the healing alveolus of this tooth and an osteolytic image (white arrows) below the mandibular canal (black arrowheads) at the lower limit of the radiograph.

Panoramic radiograph was performed (Figure 1(a)). It showed a slightly inadequate endodontic filling in the left mandibular second molar (tooth #37). No periapical bone images related to this tooth were detected. According to the patient, the pain origin was from the basilar border and not from the left mandibular second molar (tooth #37). Indeed, both axial and transverse percussions were negative. Based on these elements, diagnosis of a possible periapical complication related to the tooth #37 was, therefore, ruled out. The area around the left basilar border was unclear because the patient's head was in hypoextension when the radiograph was taken. This incorrect position led to the superposition of the hyoid bone on the basilar border, making radiological analysis impossible. A second panoramic radiograph with the head in minor hyperextension was performed, and it revealed a better exposure of the basilar border. This examination showed the presence of a well-limited homogeneous radiolucent image located below the mandibular canal on the left mandibular basilar border (Figure 1(b)). At this stage, benign left mandibular osteolytic lesion located on the cortical bone was suspected.

The patient was asked to consult his treating dentist for oral cavity rehabilitation and further endodontic treatment on tooth #37. However, the treating dentist opted for the extraction of the tooth, as it could have been the cause of the pain. Yet, no improvement was noted by the patient after the extraction of the tooth #37. Retroalveolar radiograph was then performed (Figure 1(c)). It demonstrated a healing alveolar bone, but it also revealed an osteolytic image at the basilar border, previously described in Figure 1(b).

**Figure 2:**
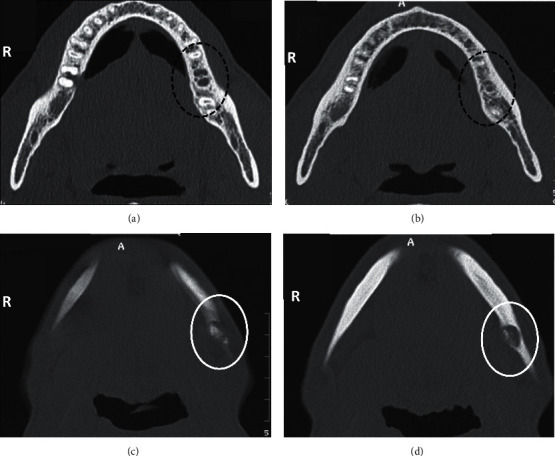
Mandibular axial computed tomography sections in bone window after extraction of the left mandibular second molar (tooth #37). (a) and (b) Sections at the alveolar bone of the tooth #37 showing the absence of bone lesions (black dashed circle). (c) and (d) Sections through the mandibular basilar border allowing the visualization of a mixed image at the region of the left basilar border of 1 cm long axis (white circle).

**Figure 3:**
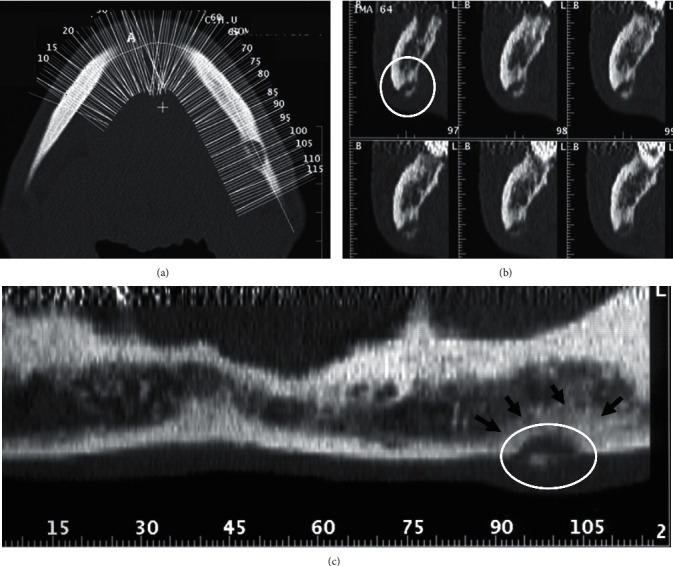
Mandibular DentaScan showing a well-limited mixed image on the left basilar border (white circle) and osteocondensation at the image periphery (black arrows). (a) Mandibular reference axial section. (b) Oblique reconstructions perpendicular to the curvature of the mandibular arch. (c) Panoramic reconstruction parallel to the curvature of the mandibular arch.

Computed tomography (CT) scan (Figures 2 and 3) was performed, and it revealed the presence of a mixed image, with a size not exceeding 1 cm on the long axis within the left mandibular cortical bone (Figures 2(c), 2(d), and 3(b)). This image had a central hyperdense area of bony nature with a surrounding hypodense halo. A zone of osteocondensation was revealed in the image periphery (Figure 3(c)). CT scan confirmed the absence of bony lesions in the alveolar bone periphery of the previously extracted tooth (Figures 2(a) and 2(b)).

Faced with this clinical presentation involving nocturnal pain and a well-limited, infracentimetric, and mixed osteolytic image with nidus and peripheral osteocondensation, diagnosis of benign osteogenic tumor of osteoid osteoma type was made.

The patient was operated under general anesthesia using exobuccal approach. An incision over the left mandibular basilar border and full-thickness flap detachment was made to provide direct access to the lesion. Curettage and esthetic sutures were then performed. The anatomopathological examination confirmed the diagnosis of osteoid osteoma. Indeed, this revealed irregular bony trabeculae lined with osteoblasts. The bony trabeculae showed varying degrees of calcification. The stroma was fibrocellular and strongly vascularized.

The patient consulted after 15 days for sutures removal. The one-month check-up revealed good healing of the wound. The outcome was favorable, and the pain disappeared.

## 3. Discussion

First described by Jaffe in 1935, osteoid osteoma is a benign osteoblastic tumor, not related to an infectious phenomenon. It frequently develops in long bones, but it very rarely occurs in the head and neck [7]. A review of the literature was carried out from the MEDLINE database via PubMed interface. The main MeSH term used was osteoid osteoma. Other MeSH terms, such as facial bones, mouth, jaw, tooth, maxilla, and mandible, were used. A total of thirty-three articles were selected, describing thirty-five cases of osteoid osteoma located in the maxillofacial region. The majority of these clinical cases reported osteoid osteoma in the mandible, mainly in the mandibular bodies. To the best of the authors' knowledge, cases of osteoid osteoma located precisely in the mandibular basilar border, as described in the present study, are very rare.

The aforementioned articles were published between 1951 and 2020. The small number of published clinical cases could be due mainly to the rarity of these lesions in the craniofacial region and the difficulty of diagnosing this benign tumor.

Correct diagnosis of this tumor requires clinical and radiological investigation. The predominant clinical feature is localized pain. The pain is often dull and boring, continuous, or intermittent. It characteristically worsens at night, and it is relieved via non-steroidal anti-inflammatory drugs (NSAIDs), which is consistent with the finding in the present case. Various theories have been advanced to explain the precise nature of the pain and its tendency to worsen at night. Some authors believe that prostaglandins, especially prostaglandin E2, play a major role in pain worsening at night. Indeed, the nidus of osteoid osteoma contains high concentration of prostaglandin E2 and prostacyclin, which explains the pain and the good response to aspirin and other NSAIDs. Other authors believe that the rich vascularity brings about innervations of the free nerve endings or direct irritation of the nerve fibers, leading to pain and tenderness [8, 9].

Regarding radiological diagnosis, standard radiography (panoramic or retro-alveolar image) remains the first-line examination. In the present case, the patient's wrong position did not allow to visualize the image and to establish the right diagnosis. This shows the importance of mastering the technique when performing panoramic radiography. In the present case, the region of the posterior mandibular basilar border coincided exactly with the projection of the hyoid bone on this site. Standard radiography must be complemented with three-dimensional imaging (computed tomography scanner or cone-beam computed tomography [CBCT]), which is the reference imaging in the diagnosis of osteoma osteoid. In dentistry, such cases are underdiagnosed since these small lesions could be missed on panoramic radiograph because of the complex anatomic site of the jaws. Indeed, CT scan or CBCT are useful imaging modalities for the diagnosis of lesions having small size. CT scans are currently the best radiographic examination for the diagnosis of osteoid osteoma. Indeed, they allow to show more details regarding the relationship between the tumor and the adjacent structures compared to conventional radiography. Furthermore, sclerotic changes of the adjacent bone and the periosteal reaction are also well demonstrated. Bone scintigraphy is useful for an early diagnosis and for guiding more precise complementary examinations. Magnetic resonance imaging (MRI), which has the advantage of being non-irradiating, is indicated when the medullary bone is affected. It is also indicated to guide diagnosis when other imaging techniques are not conclusive [8, 10]. In the present case, osteoid osteoma was diagnosed using CT scans, which not only revealed the exact dimensions and extent of the lesion, but also provided better surgical planning.

Radiographically, the typical nidus of osteoid osteoma is a round or ovoid radiolucency, less than 1 cm in diameter, having a small or no radiopacity in the center, surrounded by reactive sclerosis of the bone, and sometimes with thick homogenous periosteal reaction [11]. Osteoid osteoma is classified into three types according to the radiographic location of the nidus. It can be cortical, medullary, or subperiosteal. Cortical osteoid osteoma is the most common type, and it is usually located within the center of the sclerosis. Medullary osteoid osteoma accounts for about a quarter of the lesions, and it varies from mild to moderate osteosclerosis. Subperiosteal osteoid osteoma is less common. It appears as a soft tissue mass, adjacent to the affected bone, and it produces almost no reactive sclerosis [10].

In the present case, the axial and coronal sections of the scanner allowed to detect the radiolucent nidus with very small radio-opacity in the center and the peripheral osteosclerosis. In the present case, osteoid osteoma was located in the cortical bone.

Histologically, Jaffe described the nidus of osteoid osteoma as a hard osseous core composed of densely set trabeculae of newly formed bone, which is atypical. Jaffe defined the initial notable changes in this tumor as an increased vascularization and destruction with replacement by new atypical bone following the resorption of the demolished tissue. The stroma consists of osteogenic connective tissues, including multiple blood channels [6]. No cellular atypia or pleomorphism is present in the tumor cells. The histological appearance of osteoid osteoma varies depending on the age of the lesion and its site. Huvos distinguished three different stages of osteoid osteoma evolutionary modification. Initially, dense osteoblasts are proliferating actively in a highly vascularized stroma, followed by deposition of osteoid matrix between the osteoblasts in the intermediate phase. In the mature stage, osteoid is transformed into well-calcified compact trabeculae of atypical bone, which are neither typically woven nor lamellar [12, 13].

According to the classification of tumors having odontogenic and non-odontogenic origins, osteoid osteoma belongs to the group of non-odontogenic tumors. In fact, non-odontogenic tumors of the jaws are rare, and they are often badly understood, resulting in delayed diagnosis and treatment. Differential diagnosis arises mainly with osteoblastoma, ossifying fibroma, fibrous dysplasia, central giant cell granuloma, osteoma, and chronic osteitis with sequestrum [14]. All of these tumors are characterized by similar clinical, radiological, and histological features. Knowing how to differentiate osteoid osteoma from other tumoral lesions is, therefore, essential.

The main differential diagnosis of osteoid osteoma is osteoblastoma. Both of them share many clinical, radiological, and even histological features. Similar to osteoid osteoma, the symptoms in osteoblastoma may be absent or may manifest as pain and tenderness. However, they do not have the characteristic feature of night pain that improves with aspirin and other NSAIDs, which is typical of osteoid osteoma [3]. Unlike osteoid osteoma, osteoblastoma has a larger size of >2 cm and up to 10 cm in aggressive patterns. They do not manifest surrounding reactive osteosclerosis, with the medullary bone of axial skeleton being affected in the majority of cases [7]. Besides, it is reported that osteoid osteoma does not recur after complete removal of the nidus, whereas recurrence of osteoblastoma after complete resection is reported in several cases [11]. Despite all these differences between the two entities, some authors suggest that osteoblastoma and osteoid osteoma are anatomic variants of the same osteoblastic-origin tumor, and separation of these two tumors is, therefore, not necessary [15]. In a study carried out in 2011, Barlow et al. proposed to reclassify osteoid osteoma and osteoblastoma into a single entity, demonstrating that both of them share new identical histopathological and immunohistochemical features [7, 16]. Although ossifying fibroma has some features that are identical to osteoid osteoma, including the clinical and radiographical features, it is usually asymptomatic. It grows to a large size with no nidus and can cause resorption and displacement of teeth [17, 18]. Fibrous dysplasia is a lesion having radiolucent and ground glass appearances in the early and mature stages, respectively, which was not the case in the present study. Giant cell granuloma is distinguished based on the presence of uneven distribution of multinucleated giant cells in the surrounding stroma. This finding was not present in this case [8, 14]. Concerning osteoma, benign bone tumor is usually asymptomatic or not very painful. It does not present a nidus or a surrounding densely reactive bone, which is a characteristic feature of osteoid osteoma. Differentiation of osteoid osteoma from osteosarcoma, especially low-grade types, is important. Osteosarcoma was eliminated because the features of this lesion were compatible with a benign lesion with regard to the demarcated radiographic borders and the lack of cytological polymorphism, cellular atypia, or abnormal mitotic figures [15]. Radiologically, the finding may suggest chronic osteitis with the formation of sequestrum. However, confrontation with the clinical features does not reveal any indication in favor of this diagnosis. To avoid confusion between osteoid osteoma and similar bone pathoses, the treating practitioner should have clear concept and keen observation skill. Wrong diagnosis can be harmful to the patient as reported in the present case. The diagnosis suspected by the treating dentist was in favor of an eventual periapical complication related to the tooth #37 during endodontic treatment. The dentist wrongly proceeded to the extraction of this tooth. This decision was incorrect because of the absence of clinical and radiological signs supporting this diagnosis. Rigorous interrogation, followed by complete clinical examination, and appropriate radiological assessment are, therefore, required to make correct diagnosis. Confrontation of the radio-clinical semiology elements leads to a good diagnostic orientation and consequently to an adequate treatment.

In the literature, complete excision is the treatment of choice. Spontaneous pain disappears immediately after the surgery, as reported in the present case [19]. Recently, minimally invasive interventions, such as CT, MRI-guided radiofrequency ablation, and CT-guided laser photocoagulation have been advanced [7].

### 3.1. Key Clinical Message

Correct diagnosis of osteoid osteoma is based on the elements of clinical semiology together with the imaging findings. A wrong radiological technique or a misinterpreted radio-clinical approach inevitably leads to an incorrect diagnosis and treatment. To confirm diagnosis, the anatomopathological examination is required.

## Data Availability

Data supporting this research article are available from the corresponding author or first author on reasonable request.
